# Statistical stability and set size exert distinct influences on visual search

**DOI:** 10.3758/s13414-019-01905-2

**Published:** 2019-11-26

**Authors:** Jennifer E. Corbett, Jaap Munneke

**Affiliations:** 1 College of Health and Life Sciences, Division of Psychology, Brunel University London, MJ-122, Kingston Lane, UB8 3PH, Uxbridge, London, UK; 2 Centre for Cognitive Neuroscience, Brunel University, London, UK

**Keywords:** Perceptual averaging, Visual search, Statistical stability, Attention

## Abstract

Despite continuous retinal chaos, we perceive the world as stable and complete. This illusion is sustained over consecutive glances by reliance on statistical redundancies inherent in the visual environment. For instance, repeating the average size of a collection of differently sized items speeds visual search for a randomly located target regardless of trial-to-trial changes in local element size (Corbett & Melcher, 2014b). Here, we manipulate set size to investigate the potential role attention may play in these facilitative effects of statistical stability on visual search. Observers discriminated the left or right tilt of a Gabor target defined by a unique conjunction of orientation and spatial frequency in displays of Gabors with a stable or unstable mean size over successive trials. When set size was manipulated over sequences of successive trials, but held constant within a given sequence in Experiment 1, we observed distinct effects of statistical stability and attention, such that participants made faster correct responses as a function of stability and slower correct responses as a function of increasing set size. Replicating these main effects in Experiment 2, when set size was always unstable, provided converging evidence for discrete influences of statistical stability and attentional contributions to visual search. Overall, results support the proposal that our stable impressions of the surrounding environment and our abilities to attend salient events within that environment are distinctively governed by inherent statistical context and attentional processing demands.

How does the limited capacity visual system mediate between the needs to perceive the world as stable and complete and to detect salient objects and events? Despite being restricted to representing only a few objects in detail at any given time, the visual system capitalizes on the inherent regularity in the surrounding environment by encoding efficient statistical representations of groups of similar objects. For example, whereas observers cannot reliably recall whether a test circle was present in a previously viewed set of objects, they are able to determine whether it was larger or smaller than the mean size of the entire set (e.g., Ariely, 2001). This superior representation of average versus individual object properties suggests that the visual system represents sets of objects in a fundamentally different manner than it encodes individual object properties. Furthermore, these statistical representations persist under conditions that prohibit the detailed encoding of individual objects (Chong & Treisman, 2005; Choo & Franconeri, 2008; Corbett & Oriet, 2011; Lanzoni, Melcher, Miceli, & Corbett, 2014; Leib, Landau, Baek, Chong, & Robertson, 2012; Parkes, Lund, Angelucci, Solomon, & Morgan, 2001), and transfer across eye movements and different spatial frames of reference (Corbett & Melcher, 2014a; Corbett & Song, 2014), providing an anchor for stable perception despite the continuously changing retinal image.

Corbett and Melcher (2014b) proposed that statistical representations stabilize perception by increasing the predictability and reducing the redundancy of visual input, such that repeating the global statistical properties of a scene over successive displays facilitates visual search. When observers searched for a tilted Gabor target embedded in a random location in displays of horizontally oriented Gabors, they were faster to correctly determine whether the target Gabor was tilted to the left of right of vertical when the mean size of the background Gabors remained constant over several consecutive trials compared with when the background mean size changed on every successive display. Importantly, despite the individual Gabors changing on each display, the stability of their global mean size facilitated observers in searching for the randomly located target. In fact, Chetverikov, Campana, and Kristjánsson (2016, 2017) have since demonstrated that the visual system accumulates statistical information via internal probability density functions that are specifically tuned to background regularities over time. Taken together, these findings strongly suggest that the statistical redundancy of the surrounding environment governs the manner in which observers are able to search for salient information.

In general, the limited-capacity attentional mechanisms responsible for mediating visual search are subject to set size effects, such that when targets and background information share more than one feature, the time observers require to find the target increases linearly with the number of background items (e.g., Treisman & Gelade, 1980; Treisman & Gormican, 1988; Wolfe, Cave, & Franzel, 1989). Although these set size effects are pervasive in top-down perception, the relationship between set size and summary statistical representations remains unclear. On the one hand, several studies have found that set size does not affect mean size representations. For example, observers discriminated which of two single circles was larger with similar precision as they discriminated which of two patches of 12 differently sized circles had the larger mean size (Chong & Treisman, 2003), and were similarly precise when discriminating whether a test circle was larger or smaller than the mean size of a set regardless of whether sets were comprised of four, eight, 12, or 16 circles (Ariely, 2001). These findings suggest that limited capacity attentional mechanisms involved in visual search and detailed representation of individual objects function independently from and in a qualitatively different manner than mechanisms responsible for summarizing collections of objects and representing the statistical properties of background information (e.g., Ariely, 2001).

On the other hand, several studies have reported that set size affects the precision of summary statistical representations. In most cases, precision decreases in line with typical set size effects (Ji & Pourtois, 2018; Marchant, Simons, & de Fockert, 2013; Utochkin & Tiurina, 2014), pointing to the involvement of a serial or “inefficient” process requiring focused attention. These findings also raise the possibilities that set size may modulate the beneficial effects of background statistical stability we have previously reported (Corbett & Melcher, 2014b; Lanzoni et al., 2014), or that both mechanisms may work in a complementary manner, such that statistical stability may facilitate visual search performance by modulating set size effects. However, Robitaille and Harris (2011) found that observers became faster at determining whether a target bar was more horizontal or vertical than the mean orientation of a set of bars as set size increased, while still showing typical inefficient search RT patterns (e.g., Wolfe, 1998) when simultaneously performing a present or absent search task for a 45^o^-oriented bar within the to-be-summarized sets. In line with proposals that summary representations are constructed under a distinct distributed mode of attention that operates in parallel, efficiently over the entire display (e.g., Chong & Treisman, 2005), these findings provide additional support for the idea that set size and background statistical stability will have independent influences on visual search performance.

In an effort to better understand the potential role attention may play in the facilitative effects of statistical stability on visual search we have previously reported (Corbett & Melcher, 2014b; Lanzoni et al., 2014), we manipulated set size in a modified version of Corbett and Melcher’s (2014b) paradigm where the mean size of displays of Gabors patches remained stable or became unstable over time, and observers searched for a randomly located target. One possibility is that building background statistical stability allows for more attentional resources to be allocated to visual search, such that set size effects are attenuated and search slopes become shallower. Alternatively, statistical stability may facilitate visual search independently of attention, such that set size has a consistent effect on search time but search is overall faster compared with search in the absence of statistical stability. A final possibility is that statistical stability eliminates set size effects. However, this seems highly unlikely given that almost all studies of set size effects in visual search have used displays with consistent statistical background information.

## Experiment 1

In Corbett and Melcher’s (2014b) original study, observers searched for an orientation singleton Gabor target among 64 horizontally oriented Gabors and indicated whether the target Gabor was tilted to the left or right of vertical while the background Gabors either remained stable for sequences of five to eight successive trials (stable blocks) or changed on every trial (unstable baseline blocks). However, any independent contributions of statistical stability and set size would be masked if we adopted this exact design in the present investigation. Specifically, the first trial of stable and unstable sequences should yield similar reaction times, as these trials are only defined as stable or unstable by their relationship to subsequent trials. Therefore, we modified the original paradigm such that each sequence of trials began with a set of stable baseline trials with the same, repeated mean size, followed by either an additional series of stable trials (stable sequences) or a series of unstable trials, each with a different mean size (unstable sequences). In addition, we varied the set size for each type of sequence while holding set size constant over individual sequences. If statistical stability and attention have distinct influences on search, then we should only observe a main effect of stable versus unstable sequences and a main effect of set size, whereas if stability and set size effects on visual search are not independent, we should find an interaction between these factors.

### Methods

#### Participants

Nineteen Bilkent University students were tested in Experiment 1 (eight females, mean age = 21.7 years). All had normal or corrected-to-normal vision and voluntarily participated in the experiment in exchange for monetary compensation or course credit. All experimental procedures and protocols were approved by Bilkent University’s Ethics Committee. We based our sample size on similar sample sizes of 16 participants in the original study of statistical stability and visual search (Corbett & Melcher, 2014b), which is greater than the typical number of participants in studies of visual search (e.g., Treisman & Gelade, 1980).

#### Task

On each trial, participants searched an array of heterogeneously sized Gabors for a target Gabor with a high spatial frequency that was tilted from vertical and indicated whether it was tilted left or right of vertical. If the target was tilted to the left, they pressed the left arrow key on a computer keyboard, and if it was tilted right, they pressed the right arrow key.

#### Apparatus

An HP PC was used to present stimuli on a 21-in. NEC monitor at a resolution of 1,600 × 1,200 pixels and a 60-Hz refresh rate. MATLAB (Version 2016b) in conjunction with Psychophysics Toolbox (Brainard, 1997; Pelli, 1997) controlled all the stimulus presentation, response, and data collection functions. Participants were seated approximately 57 cm from the center of the monitor, such that one degree of visual angle corresponded to 37 pixels.

#### Stimuli and procedure (Fig. 1)

Stimuli were displays of 10, 20, or 30 (set size) Gabor patches presented at a Michelson contrast of 28%. On each trial, Gabors were randomly arranged in 49 possible locations across the entire screen within an imaginary 7 × 7 grid. Each individual stimulus location was jittered randomly in the *x* and *y* directions by 10 pixels on every trial. Whereas participants in the original study by Corbett and Melcher (2014b) searched for an orientation singleton target in displays of 64 Gabors, we used a conjunction search in the present study to guard against the possibility of “pop-out” search that could especially confound our results at smaller set sizes. Observers searched for a single high spatial frequency Gabor target tilted 45^o^ from vertical among high spatial frequency horizontally oriented Gabors and low spatial frequency 45^o^-tilted Gabors. Specifically, a random 50% of the Gabors on each trial had individual orientations randomly selected from 84^o^ and 96^o^ of tilt from vertical in 1^o^ steps and a spatial frequency of 8 cycles per degree (cpd). The remaining half of the Gabors had individual orientations randomly selected from ±45^o^ and a spatial frequency of 5 cpd except the target, which was tilted randomly ±45^o^ on each trial with a spatial frequency of 8 cpd (the target was defined by the conjunction of an oblique orientation and high spatial frequency). On each trial, the sizes of the individual Gabors were drawn pseudorandomly from a normal distribution with one of four mean sizes (1.5^o^, 2^o^, 2.5^o^, or 3^o^) and a standard deviation of 0.25^o^. The target Gabor was always 2.25^o^ (the grand mean).

**Fig. 1:**
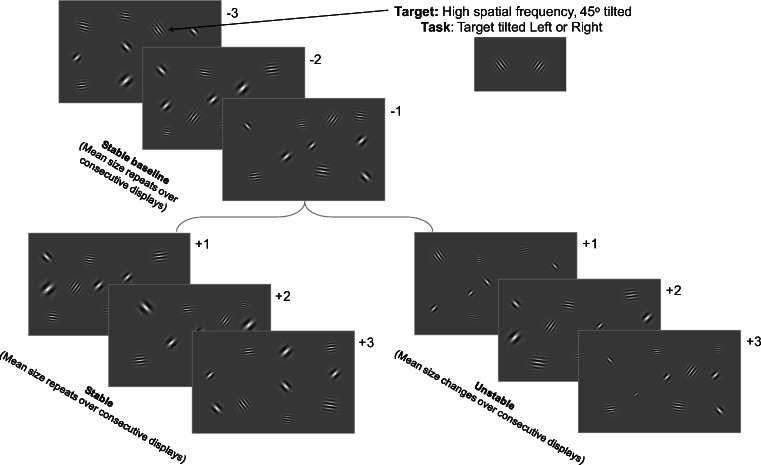
Observers searched displays of high spatial frequency horizontally oriented Gabors and low spatial frequency 45^o^-tilted Gabors for a unique high spatial frequency 45^o^-tilted Gabor target. In “stable” sequences, the set size and mean size of the background Gabors remained constant over six successive trials, whereas in “unstable” sequences, set size remained constant, but mean size changed after the first three baseline trials in each sequence

Trials were organized into stable or unstable sequences. Each sequence was composed of six trials. Each sequence began with a stable baseline of three successive trials that had the same mean size. In stable sequences, the remaining three trials also repeated the same mean size. In unstable sequences, each of the remaining three trials had a different pseudorandomly selected mean size such that no display repeated the mean size of the previous trial. The order of stable and unstable sequences was randomized within each block such that half of the sequences were stable. Trials were presented without interruption between sequences so participants were never aware of the transition between stable and unstable sequences. Set size was pseudorandomly manipulated over sequences, but remained fixed throughout a given sequence in Experiment 1. Importantly, only the mean size of the entire array of 10, 20, or 30 Gabors remained stable in the stable baseline portion of unstable sequences and the entire duration of stable sequences; the sizes of the individual Gabors (except the target) changed randomly on each trial and the spatial frequency of the individual Gabors changed pseudorandomly as previously detailed on each trial. Each block contained two sequences of each of the six different combinations of stability (stable, unstable) × set size (10, 20, 30). Each participant performed 12 blocks of 12 sequences each for a total of 144 sequences (of six trials each for a total of 864 trials per participant).

Each trial began with the presentation of the 10, 20, or 30 Gabors. Participants were instructed to search the display for the only Gabor that was both high spatial frequency and tilted from vertical, and then to use the computer keyboard to indicate whether this target was tilted to the left or right using the corresponding arrows. Stimuli remained visible on the screen until a response was given. Before the experiment, participants were given one block of 12 practice sequences (72 trials) to familiarize them with the task. Data from these practice blocks were not analyzed. Given that participants could view each display as long as they wanted, they were required to complete the practice block and all experimental blocks with at least 90% accuracy. One participant (not included in the above description of participants or in any analyses) was dismissed from the experiment after the failing to achieve this 90% accuracy criterion during the practice block. In all practice and experimental blocks, participants were instructed to respond as quickly and accurately as possible. Also, participants were never given specific instructions about how to search displays (e.g., starting from a central position), so as not to confound any potential effects of stability by imposing search strategies.

### Results and discussion

**Fig. 2:**
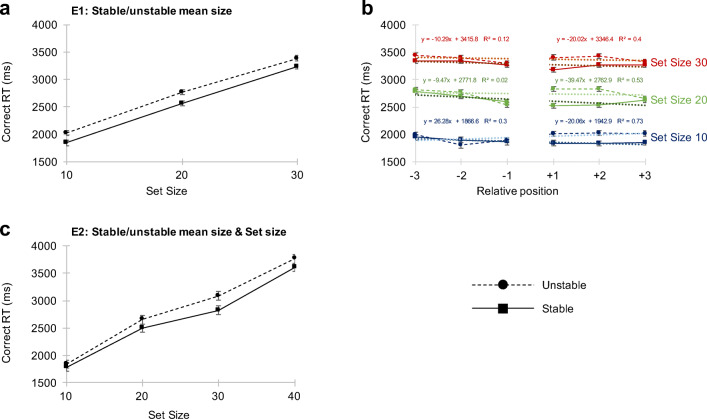
Results. In Experiment 1, (**a**) when set size remained stable over six consecutive trials, but mean size either remained stable or became unstable for the three last trials in a given sequence, stability facilitated search independently from the increase in RTs as a function of increasing set size. **b** This effect was observed for sequences with each of the three possible set sizes. In Experiment 2, (**c**) the same pattern of results was obtained such that mean size stability facilitated search independent of increasing RTs for increasing set sizes even when set size became unstable. Equations in panel **b** represent the linear fits, and *R*^2^ values of the corresponding linear trend lines in each unstable (left) and stable (right) condition. In all panels, error bars represent the 95% within-subjects confidence intervals for the corresponding two-way interactions (Loftus & Masson, 1994)

In all analyses, we only included trials with correct responses. Response times (RTs) were first pruned using a two standard deviations from the mean cutoff, calculated separately for each participant in each of the six combinations of stability × set size. Less than 6% of all trials (including trials with incorrect responses) were trimmed for any given participant in Experiment 1. As we were primarily interested in the effects of statistical stability and set size, we first analyzed only the last three trials in each sequence (when the mean size of the Gabors either repeated as in the sequence’s three previous baseline displays or became unstable relative to the constant mean size of the Gabors in the three, preceding baseline displays). As illustrated in Fig. 2a, A 2 (stability) × 3 (set size) repeated-measures within-subjects ANOVA on participants’ resultant average RTs revealed a main effect of stability, *F*(1, 18) = 14.55, *MSE* = 63402.68, *p* = .001, η_p_^2^ = 0.447, and a main effect of set size, *F*(2, 36) = 125.65, *MSE* = 144392.15, *p* < .001, η_p_^2^ = 0.875, but, crucially, no interaction, *F*(2, 36) = 0.221, *MSE* = 39342.42, *p* = .803, η_p_^2^ = 0.012. We further analyzed the null interaction effect using Bayesian information criteria (Wagenmakers, 2007), comparing the fit of the data under the null hypothesis that included main effects of both stability and set size and the alternative hypothesis with the addition of the interaction between these two main factors. This analysis gives the likelihood of the data arising from a main effect model compared with a model with an interaction. The estimated Bayes factor of 6.494 suggested that the data were 6.494 times more likely to occur under a model including the interaction than a model including only the main effects stability and set size (with 3.767% error).

As further illustrated in Fig. 2b, there were similar patterns for all three set sizes, such that the benefits of statistical stability continued when the mean size remained stable but search RTs increased sharply when stability was broken. These conclusions are further supported by the results of an ANOVA on participants’ two standard deviation normalized correct response-time data from all six trials in a given sequence (the first three stable baseline trials plus three subsequent stable/unstable trials), which confirmed significant effects of stability, *F*(1, 18) = 9.515, *MSE* = 183494.29, *p* = 0.006, η_p_^2^ = 0.346, and set size, *F*(2, 36) = 131.06, *MSE* = 869712.1, *p* < .001, η_p_^2^ = 0.879, but also revealed significant main effects of trial number in sequence, *F*(5, 90) = 2.745, *MSE* = 93914.55, *p* < .024, η_p_^2^ = 0.132, and an interaction between stability and trial number in sequence, *F*(5, 90) = 3.916, *MSE* = 73392.54, *p* < .003, η_p_^2^ = 0.179. Further support is given by the findings of a linear trend of trial number in sequence in the stable response times, *F*(1, 18) = 7.673, *MSE* = 91392.81, *p* = .013, *η*_p_^2^ = 0.299, that was not observed for the unstable response times, *F*(1, 18) = 0.051, *MSE* = 91547.72, *p* = .823, η_p_^2^ = 0.003. Taken together, the patterns of results obtained in Experiment 1 provided the first evidence that attention (as indexed by set size effects) and statistical stability independently influence visual search.

## Experiment 2

Although the results of Experiment 1 suggested that statistical stability and attention exert independent influences on visual search performance, displays in Corbett and Melcher’s (2014b) original study always contained 64 items, and displays in Experiment 1 of the present study always maintained the same set size throughout each stable or unstable sequence of trials. Therefore, we varied set size on each nonbaseline trial in Experiment 2 to confirm that the stability of mean size would still facilitate search even when set size became unstable over consecutive trials. Importantly, we did not design Experiment 2 as a test of whether set size effects change over time when the stability of the background information is held constant, as this is what has typically been done in paradigms used to measure set size effects on visual search (e.g., Treisman & Gelade, 1980; Treisman & Gormican, 1988; Wolfe et al., 1989). Instead, we aimed to test a potentially stronger manipulation than simply varying stability with set size constant across a given sequence as in Experiment 1: whether the stability of mean size would still facilitate search when set size continuously varied.

### Method

#### Participants

Nineteen Bilkent University students that had not participated in Experiment 1 were tested in Experiment 2 (10 females, mean age = 21.4 years). All had normal or corrected-to-normal vision and voluntarily participated in the experiment in exchange for monetary compensation or course credit. One additional participant (not included in the above description) was dismissed from the experiment for failing to perform with above 90% accuracy in the practice block.

#### Task, apparatus, stimuli, and procedure (Fig. 3)

All aspects of Experiment 2 were identical to Experiment 1, with three key exceptions. First and foremost, instead of manipulating set size over sequences of trials as in Experiment 1, set size was pseudorandomly selected on each nonbaseline trial in Experiment 2 such that two successive nonbaseline trials never had the same set size. As a result, only the mean size of the items remained stable over stable sequences, with set size and mean size stable in baseline trials for both stable and unstable sequences. As always, individual element sizes changed on every trial. In addition to this major modification, we randomly varied the length of each stable or unstable sequence of trials such that there were between two and four baseline trials with stable mean and set sizes, followed by four trials that repeated only the mean size (stable sequences) or four trials with different consecutive mean and set sizes (unstable sequences). Randomizing the length of the baseline in this manner helped to control for any temporal patterns participants may have otherwise noticed if the number of elements in each display was always the same for three consecutive trials. Finally, we included a fourth set size of 40 items to equate the number of set sizes and mean sizes used in Experiment 2. Each participant performed eight blocks of eight sequences each for a total of 64 sequences (of six to eight trials each for a total of approximately 448 trials per participant). Each block contained one sequence of each of the eight different combinations of stability (stable, unstable) × set size (10, 20, 30, 40).

**Fig. 3:**
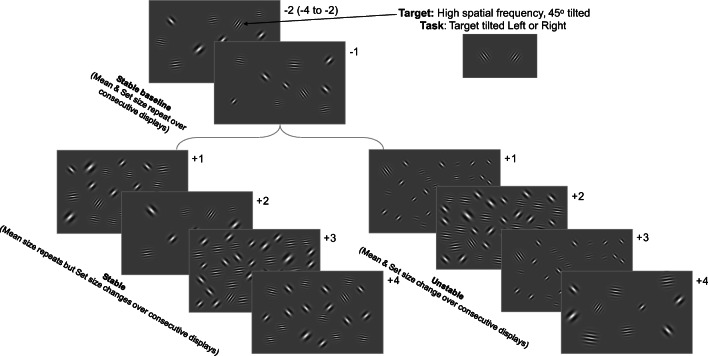
Observers searched displays of high spatial frequency horizontally oriented Gabors and low spatial frequency 45^o^-tilted Gabors for a unique high spatial frequency 45^o^-tilted Gabor target. In both the stable and unstable sequences, mean size and set size repeated over the first two to four consecutive baseline trials. Then, in the stable sequences, set size remained constant, but the mean size of the background Gabors changed on each of the four subsequent displays and in the unstable sequences, both the set size and mean size of the background Gabors changed on each of the four subsequent displays

Note that we factorially varied set size in the (stable) baseline trials of both stable and unstable sequences. In nonbaseline trials, set size was randomly determined such that each consecutive nonbaseline trial had a different set size in both stable and unstable sequences. Only mean size remained constant over successive nonbaseline trials in stable sequences. In other words, our analysis is concerned with the effects of the four different set sizes in these nonbaseline trials as a function of whether the mean size of the displays remained stable or became unstable. As such, factorially varying the set size of the baseline of each sequence helped to ensure that any carryover effects of set size from the (stable) baseline trials in each sequence would average out over the nonbaseline trials. This allowed us to restrict our analysis to the effects of stable versus unstable mean size when the number of elements in successive displays became unstable.

### Results

To confirm that the effect of stability persisted even when set size became unstable, we repeated the original analysis in Experiment 1 (see Fig. 2a) for four nonbaseline trials in each of the eight combinations of stability and set size tested in Experiment 2.^1^ We again began by normalizing each participant’s conditional response times with a two standard deviations from the mean cutoff. Less than 7% of all trials (including trials with incorrect responses) were trimmed for any given participant in Experiment 2. As illustrated in Fig. 2c, a 2 (stability) × 4 (set size) repeated-measures within-subjects ANOVA on participants’ average conditionally normalized correct RTs in nonbaseline trials again revealed main effects of stability, *F*(1, 18) = 4.627, *MSE* = 206155.33, *p* = .045, η_p_^2^ = 0.204, and set size, *F*(3, 54) = 63.055, *MSE* = 365348.979, *p* < .001, η_p_^2^ = 0.778, but no interaction between these factors, *F*(3, 54) = 0.64, *MSE* = 114186.1, *p* = .593, η_p_^2^ = 0.034. We again performed a Bayesian analysis of the null interaction effect by comparing the fit of the data under a null model including the two main effects and an alternative model adding the interaction. The estimated Bayes factor of 10 suggested that the data were 10 times more likely to occur under a model including the interaction than a model including only the main effects stability and set size (with 2.108% error). These patterns of results paralleled the independent effects of statistical stability and attention suggested by the results of Experiment 1, providing further evidence that the stability of the mean size of the displays facilitated participants in finding the randomly located target amongst constantly changing local information, regardless of the increase in search times for displays with more items.

## General discussion

The present investigation provides converging evidence for distinct influences of statistical stability and attention on visual search. Building on previous reports of speeded search for randomly located targets in arrays of elements with repeated versus changing mean sizes (Corbett & Melcher, 2014b), we manipulated set size to index the extent to which these statistical stability effects may depend on attentional resources well known to influence visual search performance (e.g., Treisman & Gelade, 1980; Treisman & Gormican, 1988; Wolfe et al., 1989). In Experiment 1, we observed distinct effects of stability and set size when set size was constant and the mean size of elements either remained stable or became unstable, with faster responses for stable versus unstable trials and slower responses as a function of increasing set size. These effects of statistical stability were evident over the evolution of six-trial sequences in each of the three set sizes, further suggesting that stability and attention exert discrete influences on search. Experiment 2 confirmed that the observed effects of stability were truly due to the stability to of mean size of elements over successive displays that could not otherwise be accounted for by the repeated set sizes used in Experiment 1 and all previous investigations. As in Experiment 1, mean and set size were constant for baseline trials in each sequence. However, only mean size remained stable in nonbaseline trials of stable sequences, whereas mean and set size changed on every consecutive unstable nonbaseline trial. The results of Experiment 2 again revealed distinct contributions of mean size stability and set size in the nonbaseline trials, demonstrating that the statistical stability of background information still facilitated search even when set size became unstable over consecutive trials. Taken together, these results do not support the hypothesis that statistical stability and attention interactively affect visual search performance, but instead suggest that these factors exert independent influences on observers’ abilities to find salient targets within dynamic backgrounds.

Importantly, our conclusion that the facilitatory effects of statistical stability on visual search persist despite increasing attentional demands (as indexed by set size effects) relies not only on distinct significant main effects of stability and set size but also on the lack of a significant interaction between these two factors across two experiments. In other words, whereas there is evidence that stability and set size both exert influences on search performance, there is no evidence to support the hypothesis that these factors interactively affect search. We have attempted to further support our claims with Bayesian interpretations of the much greater likelihoods of the observed results occurring under models without versus with the interaction. Nonetheless, it is possible that our manipulations were not sensitive to some less obvious ways in which set size and statistical stability could possibly interact. Hopefully, the current study will provide a starting point, opening future investigations into the potential ways that statistical stability efficiently guides our perceptual experience.

Overall, results support the proposal that our stable impressions of the surrounding environment and our abilities to attend salient events within that environment are guided by differential influences of inherent statistical context and attentional processing demands. Although our results suggest discrete influences of attention and statistical stability on visual search, the present results do not speak directly to the standing controversy regarding the role of attention in summary statistical representations. Importantly, the set size and statistical stability effects on participants’ correct reaction times observed in the present investigation are not the same as set size effects considered in several previous studies measuring the accuracy with which observers are able to estimate the mean properties of groups of objects (Ariely, 2001; Chong & Treisman, 2003; Ji & Pourtois, 2018; Marchant et al., 2013; Robitaille & Harris, 2011; Utochkin & Tiurina, 2014). In the context of the growing literature on perceptual averaging, our results do support proposals that summary representations underlying the observed stability effects are constructed in a qualitatively different, distributed, more efficient manner than detailed representation of individual objects accomplished via the limited capacity attentional mechanisms involved in visual search (e.g., Ariely, 2001; Chong & Treisman, 2005). The present results can also be interpreted in line with proposals that summary representations are constructed during a more preattentive stage of processing before the limited capacity bottleneck (Chong & Treisman, 2005), where they can facilitate the segmentation of target objects from backgrounds prior to the actual search stage of the task (e.g., Wolfe, Oliva, Horowitz, Butcher, & Bompas, 2002). The late Anne Treisman’s significant contributions to each literature accentuate the importance of these distinctive influences of attention and summary statistical representation on visual perception.

### Open practices statement

Raw data for both experiments is publicly available on the Open Science Framework (https://osf.io/5kv64/). All stimulus presentation and analysis code is available upon request made directly to either of the authors via the provided e-mail addresses. None of the experiments were preregistered.

## Footnotes

1 Note that we did not include a similar analysis including trial number in sequence as in Experiment 1 because we designed Experiment 2 to test for a potential interaction between mean size and set size in the nonbaseline trials only, not to document the typical set size effects observed in most previous studies where mean size remains constant and only set size varies.
